# Nanoscale-Textured Tantalum Surfaces for Mammalian Cell Alignment

**DOI:** 10.3390/mi9090464

**Published:** 2018-09-13

**Authors:** Hassan I. Moussa, Megan Logan, Kingsley Wong, Zheng Rao, Marc G. Aucoin, Ting Y. Tsui

**Affiliations:** 1 Department of Chemical Engineering, University of Waterloo, Waterloo, ON N2L 3G1, Canada; h2moussa@uwaterloo.ca (H.I.M.); m3logan@uwaterloo.ca (M.L.); kingsley.wong@edu.uwaterloo.ca (K.W.); z2rao@edu.uwaterloo.ca (Z.R.); marc.aucoin@uwaterloo.ca (M.G.A.); 2 Waterloo Institute of Nanotechnology, University of Waterloo, Waterloo, ON N2L 3G1, Canada

**Keywords:** tantalum, mammalian cells, morphology, biomaterials, nanoscale

## Abstract

Tantalum is one of the most important biomaterials used for surgical implant devices. However, little knowledge exists about how nanoscale-textured tantalum surfaces affect cell morphology. Mammalian (Vero) cell morphology on tantalum-coated comb structures was studied using high-resolution scanning electron microscopy and fluorescence microscopy. These structures contained parallel lines and trenches with equal widths in the range of 0.18 to 100 μm. Results showed that as much as 77% of adherent cell nuclei oriented within 10° of the line axes when deposited on comb structures with widths smaller than 10 μm. However, less than 20% of cells exhibited the same alignment performance on blanket tantalum films or structures with line widths larger than 50 μm. Two types of line-width-dependent cell morphology were observed. When line widths were smaller than 0.5 μm, nanometer-scale pseudopodia bridged across trench gaps without contacting the bottom surfaces. In contrast, pseudopodia structures covered the entire trench sidewalls and the trench bottom surfaces of comb structures with line-widths larger than 0.5 μm. Furthermore, results showed that when a single cell simultaneously adhered to multiple surface structures, the portion of the cell contacting each surface reflected the type of morphology observed for cells individually contacting the surfaces.

## 1. Introduction

As a biomaterial [1], tantalum uses include radiopaque bone marker implants and cranioplasty plates [2]. Its alloys have shown promise as orthopedic implant materials due to their osseointegration and bone ingrowth characteristics [3,4,5]. These metal implants can be used in dense form [6,7] or in porous scaffold structures [4,8,9,10,11] for hip and knee arthroplasty [4], spine surgery [4], knee replacement, and avascular necrosis surgery [4,9]. Porous metal scaffolds are used to enhance bone tissue ingrowth and to improve stability performance. The elastic modulus and hardness of 100 nm-thick tantalum thin films are 176.1 ± 3.6 GPa [12] and 12.11 ± 0.46 GPa [12], respectively. Tantalum has a weighted surface energy of ~2.42 J/m^2^ [13], which is larger than titanium’s weighted surface energy of ~2.0 J/m^2^ [13]. Balla et al. [10] showed that human fetal osteoblast cells exhibit better cellular adhesion, growth, and differentiation performance on 73% porous tantalum compared to on titanium control samples. Furthermore, cell densities were six-fold larger on porous tantalum compared to titanium under the same culture conditions. As a result, tantalum thin films are also used to coat porous titanium [14] and carbon scaffold structures [15] to promote implant surface osseointegration and ingrowth characteristics. Although cell responses on bulk specimens are well-established, little knowledge exists about how nanometer-scale textured tantalum surfaces affect cell adhesion and morphology. This information is important as medical implant surfaces may consist of nanometer-scale topographic structures produced during the fabrication processes, for example through mechanical polishing and handling.

The mechanism of cell adhesion and the resulting morphology on different surfaces is complex, often dependent on a wide range of factors such as the protein species adsorbed on the surfaces [16,17], surface structure geometries [17,18,19,20,21], roughness [22,23,24,25,26,27], and surface energy of the substrata [22,28]. Recently, novel functional biocompatible ferroelectric materials, such as lithium niobate and lithium tantalate, have been used to manipulate cell behavior [29,30,31,32,33,34,35]. In particular, the surface charge of these materials is able to enhance osteoblast function, mineral formation [31], and create human neuroblastoma cell patterns [35]. The influences of topographic-based parallel line surface structures on cell adhesion, morphology, and behaviors have been studied by several researchers [36,37,38,39,40,41,42,43,44,45,46,47,48,49]. Some of the literature results for topography-induced morphological changes are summarized in Table 1. Substrate materials used in prior works are limited to polymers, silicon oxide, or silicon. In addition, the range of line width examined in each prior study was often restricted to within two orders of magnitude. The majority of studies thus far have been limited to effects and analysis on a micron scale. There is little information probing effects occurring at or due to sub-micron features. A driving hypothesis of the work presented here is that the range of line widths reported thus far in the literature has limited the ability to gain a full understanding of the effects of surface patterning on cell behavior. However, it is clear from Table 1 that the sensitivity of cell morphology and cell alignment as a result of surface pattern geometries, such as line and trench widths, varies significantly among the cell type and substrate material. No report currently exists regarding the behavior of mammalian cells on nano-textured tantalum surfaces, in part due to the difficulties associated with producing these metal specimens. However, tantalum is increasing in popularity as an implant material. Together with the fact that controlling cell alignment on material surfaces improves the success rate of implants [50,51,52,53], there is a need to further understand cell morphology on nano-textured tantalum surfaces.

At the core of this study on cell behavior is how a cell responds to its environment. Cellular organelle-like pseudopodia play an important role in contact guidance, focal adhesion, and motility processes. These cell behaviors are regulated by complex protein-protein interactions and pathways [55,56]. There are wide varieties of pseudopodia and their classifications are commonly based on their morphology, resulting in a sub-classification of filopodia, reticulopodia, and axopodia. These cytoplasmic projections are regulated by different molecular signal transduction pathways.

Hence, the primary objective of this work was to develop an understanding of how complex tantalum-coated nano- and micro-scale comb structures influence mammalian cell morphology and spreading mechanisms. The comb structures included parallel lines and trenches with widths in the range of 0.18 to 100 μm. This study covers more than three orders of magnitude of line/trench widths from nanometer to sub-millimeter scale and is thought to be the largest range by a single investigation to date. Tantalum was chosen for this study in part due to its broad applications in implants [4], mechanical strength [13], corrosive resistance, in vivo bioactivity, and bio-compatibility [57]. Tantalum is even surpassing titanium as a material of choice for certain applications. A secondary aspect enabled by this study was the examination of individual localized responses of cells adhering to multiple patterned tantalum structures having vastly different geometries. In this work, special attention has been given to the behavior of pseudopodia with diameters smaller than 100 nm. Morphology of adherent cells were characterized using high-angle tilted high-resolution field-emission scanning electron microscopy (SEM) and high-resolution fluorescence confocal microscopy techniques. Results showed that cell adhesion and morphology depended not only on line and trench widths but also on the depth pseudopodia penetrated into the trench space. The morphology of an individual cell that simultaneously adhered to different surface pattern structures showed that cells had significantly different localized morphologies and spreading behaviors within the context of a single cell.

## 2. Materials and Methods

### 2.1. Tantalum Comb Structures

**Figure 1:**
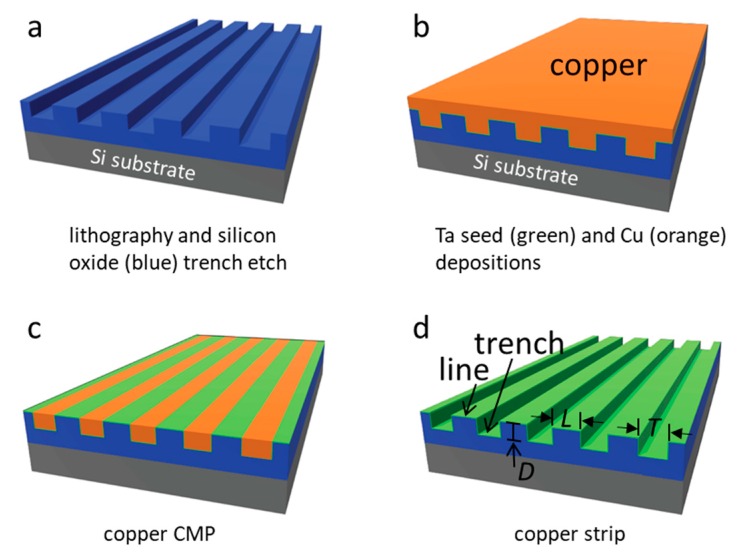
Schematic drawings illustrate the tantalum (green) comb structure fabrication method. (**a**) Patterns were transferred to the silicon oxide films using lithography and plasma etching techniques. (**b**) Tantalum seed and copper films were deposited on the etched patterns. (**c**) Excess copper was removed by using the chemical-mechanical polishing techniques. (**d**) Remaining copper was stripped with nitric acid. The comb structure contains line (L) and trench (T) of equal widths. All trenches had the same depth (D).

Tantalum thin film-coated comb structure specimens were fabricated using an advanced integrated circuit back-end-of-line processing method on 200-mm silicon wafers [58,59,60]. They were supplied by Versum Materials, LLC (Tempe, AZ, USA). The fabrication steps for these silicon-based devices are briefly summarized and illustrated in Figure 1. Parallel line comb structures with equal-width trenches (T) and lines (L) were transferred to the silicon oxide films deposited on the silicon substrate using lithography and dry etching techniques [58,59,60,61]. The rectangular-shaped comb structure areas were no smaller than 1.8 mm^2^ with widths larger than 1 mm. The tantalum seed layer and copper were deposited on these patterned surfaces, and excess copper was removed using chemical mechanical polish methods [61,62,63]. The remaining copper was stripped by submerging the specimens in ~9.4 M nitric acid for ~45 min followed by rinsing with deionized water and ethanol. This acid-stripping agent was a diluted solution from 70% nitric acid ACS Plus (Fisherbrand®, Fisher Scientific International Inc., Pittsburgh, PA, USA). The line and trench dimensions fabricated are summarized in Table 2. The trench depths (D) of all patterned comb structures were fixed at ~700 nm.

### 2.2. Cell Culture and Deposition

Detailed cell culturing techniques have been presented elsewhere [18]. Briefly, Vero cells (CCL-81) acquired from the American Type Culture Collection (ATCC, Manassas, VA, USA) were cultured in an equal volume of F12 (Corning, NY, USA) media and Corning® Cellgro™ Dulbecco’s Modified Eagle Media (DMEM). The media was supplemented with 4-mM L-glutamine (Sigma-Aldrich, St. Louis, MO, USA) and Gibco™ 10% (*v*/*v*) fetal bovine serum (FBS) by Thermo Fisher Scientific (Waltham, MA, USA). Cell culture was performed in 25 mL media under 5% CO_2_ atmosphere at 37 °C using tissue-culture-treated 175 cm^2^ flasks (Corning Falcon, Corning, New York, NY, USA). Before inoculation with cells, copper-stripped specimens were sterilized with a 70% ethanol solution for 30 s. This was followed with a Dulbecco’s phosphate-buffered saline (D-PBS) rinse. Unless otherwise noted, copper-stripped specimens were then inoculated with ~0.5 × 10^5^–~1.0 × 10^5^ cells/mL and incubated in 6-well tissue culture plates (Nunc, Thermo Scientific, Hvidovre, Denmark) at 37 °C for 0.5 to 24 h.

### 2.3. Cell Fixation and Staining Processes

All tantalum specimens with adherent cells were rinsed with a D-PBS solution after the prescribed length of incubation and fixed with a solution of 4% methanol-free formaldehyde (Sigma-Aldrich, Oakville, ON, Canada) for 1 h in ambient conditions. The fixed cells were permeabilized in a 0.1% Triton-X 100 (Sigma-Aldrich) solution for 5 min. Specimens were rinsed with PBS and blocked with 2 mL of 1% (*w/w*) bovine serum albumin (BSA) (Sigma-Aldrich). F-actin microfilament staining was conducted by soaking specimens for 1 h in the deep red CytoPainter F-Actin stain (ab112127 Abcam, Cambridge, MA, USA) solution, which was diluted by a factor of 1000 in 1% BSA. A solution of 0.4 μg/mL of the 4′,6-diamidino-2-phenylindole (DAPI, Life Technologies, Waltham, MA, USA) was used to stain the DNA (5 min). All staining processes were performed in the dark to avoid photobleaching and the specimens were rinsed twice with 2 mL D-PBS after each stain application. The final solution contained four drops of Prolong Gold anti-fade reagent (Life Technologies). Specimens were kept refrigerated at 4 °C. A Leica TCS SP5 confocal fluorescence microscope (Wetzlar, Germany) at the University of Guelph, Ontario, Canada, was used to inspect stained samples with wavelengths in the range of 436 to 482 nm (for DAPI) and 650 to 700 nm (for CytoPainter F-Actin). 

### 2.4. Scanning Electron Microscopy

Prior to the SEM inspections, formaldehyde-fixed specimens were dehydrated by soaking them successively in ethanol solutions with increasing concentration: 50%, 75%, 95%, and 100% (*v*/*v*). Specimens soaked in the 50% and 75% ethanol were kept in the solution for 10 min each. The final drying processes were completed by two 10-min soaking steps in each of the 95% and 100% solutions. Specimens were dried and then stored in a nitrogen box. Cell cross-sectioning was conducted by using a three-point bend micro-cleaving technique under ambient conditions. Cell inspection and imaging were carried out with a field-emission scanning electron microscope (SEM, Zeiss 1550, Carl Zeiss AG, Oberkochen, Germany). The accelerated voltage was maintained at 7 kV. None of the SEM specimens were coated with gold or other conducting materials.

### 2.5. Adherent Cell Alignment and Elongation Characterizations

**Figure 2:**
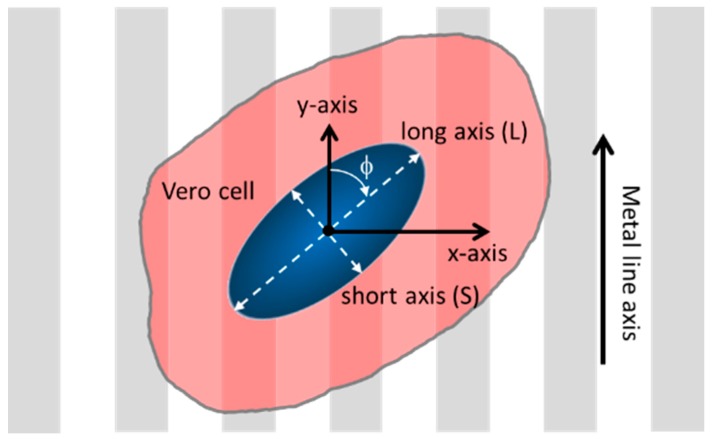
Schematic drawing of a cell on patterned comb structure and their orientation and elongation parameters.

The orientations of adherent cells were characterized by the angles (ϕ) between the long axis of the cell nuclei and the comb structure line axes, as schematically illustrated in Figure 2. The angle of a nucleus’ long axis is 90° when it is normal to the line axes (y-axis), whereas the nucleus is aligned parallel to the lines at an angle of 0°. The amount of nuclear elongation was characterized by the ratios between the dimension of the elliptical-shaped nuclei along the long (L) and short (S) axes. Elongated cell nuclei have large L/S values, whereas cells with perfect circular geometry have length ratios of 1. These parameters were manually measured using the built-in functions, Angle and Straight, of Image Processing and Analysis in Java (ImageJ) software (National Institute of Mental Health, Bethesda, MD, USA). To prevent possible influence from the edges of the patterned regions, only measurements recorded from cells that were located further than 50 μm from the perimeter were included in the analyses.

## 3. Results

### 3.1. Test Structure Characterizations

Representative 70° tilted SEM micrographs of copper-stripped test structures with varying line widths and spacing are displayed in Figure S1. The entire surface of the specimen, including sidewalls, was coated with a thin (~20 nm) conformal layer of tantalum. The micrographs revealed that all the copper had been removed and the trench side walls were vertically aligned with the substrate surfaces. Both line and trench bottom surfaces were smooth without any observable processing residues. The trenches were approximately 700 nm deep.

### 3.2. Cell Alignment and Elongation on Patterned Comb Structures

**Figure 3:**
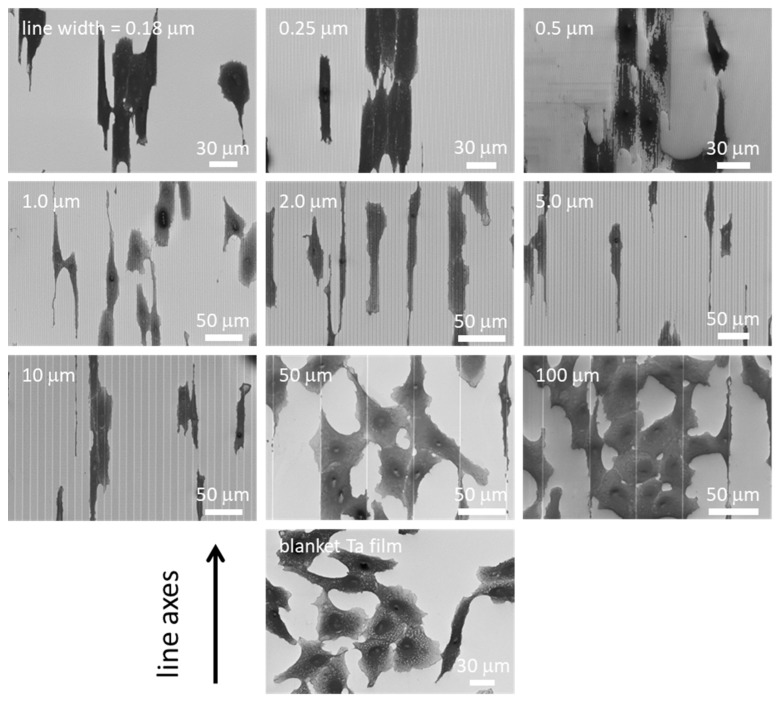
Representative top-down scanning electron microscopy (SEM) micrographs of adherent cells on different comb structures and blanket tantalum (Ta) film. Results show that adherent cells are aligned to the line axes on structures with line widths in the range of 0.18 to 10 μm. In contrast, cells on the 50 μm and 100 μm structures do not align well with the line axes—they are similar to cells randomly distributed on blanket Ta films. All cells were incubated on these specimens for 24 h.

**Figure 4:**
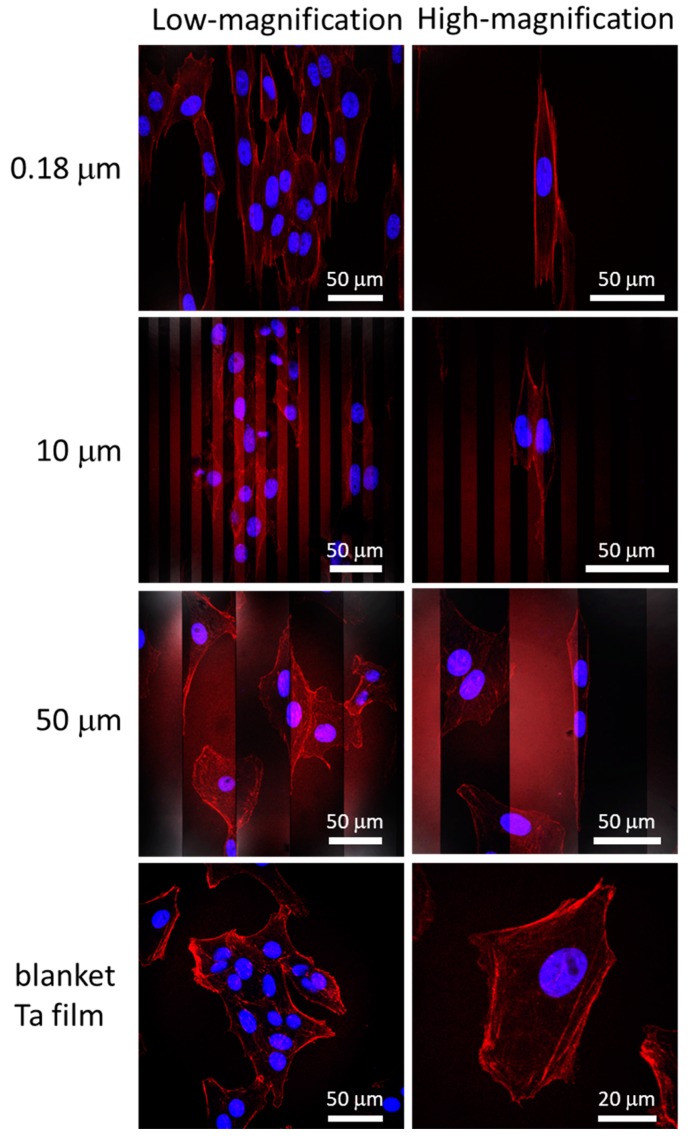
Typical fluorescence confocal micrographs of adherent cells on blanket Ta thin film and comb structures with line widths of 0.18, 10, and 50 μm. Cell nuclei appear blue (4′,6-diamidino-2-phenylindole; DAPI), whereas F-actin microfilaments appear red (fluorescent phalloidin conjugate).

Representative top-down SEM micrographs of adherent cells on the comb structures and blanket tantalum thin film surfaces are displayed in Figure 3. These cells were incubated on these comb structures for 24 h. The comb structures included alternating parallel lines and trenches of the same width. Even though patterns with line widths smaller than 1.0 μm were indiscernible due to the magnifications, the images had lines that were vertically aligned. Micrographs clearly showed that adherent cells on comb structures, having 0.18, 0.25, 0.5, 1.0, 2.0, 5.0, and 10 μm lines (L) and trenches (T) of equal widths, were elongated along the line axes. In contrast, cells on the 50 and 100 μm comb structures maintained arbitrary shapes and did not show any strong orientation preference, and exhibited a similar morphology to that on flat blanket tantalum surfaces. Cell alignment characteristics on the comb structures were also verified with fluorescence confocal microscopy techniques. Micrographs of adherent cells on comb structures with line widths of 0.18, 10, and 50 μm are displayed in Figure 4. The cell nuclei (blue) and F-actin microfilaments (red) were stained with DAPI and phalloidin conjugate, respectively. Results showed that elongated adherent cells and their nuclei were aligned with the line axes on the 0.18 and 10 μm comb structures. In contrast, the majority of cells attached on the 50 μm lines were oriented randomly, similar to those on blanket tantalum thin film surfaces. These observations confirmed that the orientation of the cell nucleus followed the overall adherent cell alignment direction. Additional fluorescence confocal micrographs of cells on 0.25, 0.5, 1.0, and 10 μm comb structures are shown in Figure S2 to demonstrate the reproducibility of the cell morphology. These micrographs show that the cell and nuclear elongation behaviors were consistent with those observed in the SEM micrographs of Figure 3.

**Figure 5:**
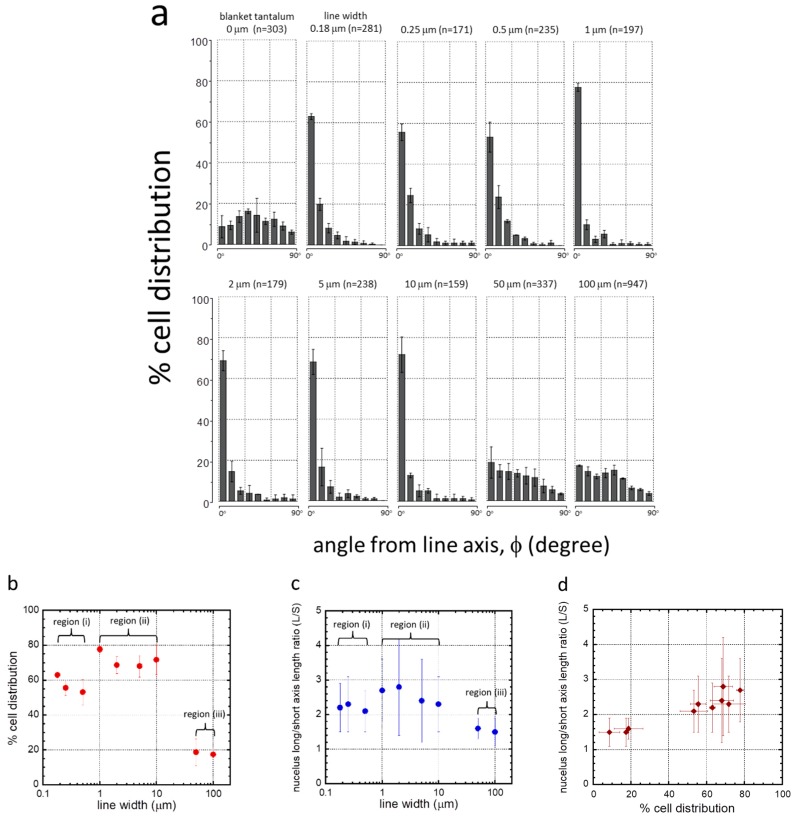
(**a**) Plots of cell orientation (ϕ) distribution in percentage on 0.18, 0.25, 0.5, 1, 2, 5, 10, 50, and 100 μm-wide line comb structures. As a comparison, this figure includes measurements from blanket tantalum films. The number of cells inspected (n) on each pattern is displayed in an individual chart. Each bar represents a 10° bin of deviations from the line axis in either clockwise or anti-clockwise directions. For example, a cell nucleus deviated from line axis of –22° would fall into the third bin of each plot. These results show that most adherent cells are aligned to the line axes on comb structures in the range of 0.18 to 10 μm. Adherent cells orientations are increasingly randomized on comb structures with line widths of 50 and 100 μm. (**b**) Percent cell distributions that aligned within ±10° of the comb structure line axes. (**c**) Plot of ratio of nucleus long and short axes as a function of comb structure line widths. (**d**) Plot of nucleus axis length ratio (L/S) as a function of percent cell distribution aligned within ± 10° of line axes.

To quantify the cell alignment and elongation behavior, the orientation of the cell’s nucleus relative to the line axis (ϕ) and its dimensions were measured. Figure 5a shows the percentage of the population of cell nuclei that oriented at various angles from the line axes. Specimens with cell nuclei randomly oriented should have an equal distribution in each bin i.e., ~11%. Error bars shown in Figure 5a,b represent one standard deviation from the results of three random groups of cell nuclei. The number of cells and the coverage density (cells/mm^2^) of each comb structure are reported in Table 2. Results show that the adherent cells were randomly oriented on the blanket tantalum thin film surfaces with no distinct preferred nuclear orientation. In contrast, cells on the 0.18 to 10 μm comb structures favored alignment parallel to the lines. 

To highlight this behavior, the population of cells oriented within ±10° of the line axes is plotted as a function of line width in Figure 5b. Results indicate that there were three possible alignment regimes based on line widths: (i) 0.18 to 0.5 μm, (ii) 1 to 10 μm, and (iii) 50 to 100 μm. In region (i), ~53% to ~63% of the adherent cell population were oriented within the 10° angular range. As line widths increased to between 1 and 10 μm in region (ii), a larger portion of cells, ~68% to ~78%, were aligned with the line axes. Increase in line widths beyond 50 μm, region (iii), led to a sharp decline in cell alignment performance with fewer than 19% of the cell population oriented parallel to the line axes.

The influence of line width on nuclear elongation was also characterized by comparing the average axis length ratio (L/S) of the cell nuclei on various comb structures, as shown in Figure 5c. Results showed that cell nuclei were significantly elongated when cells were adhered to comb structures with line widths in the range of 0.18 to 10 μm. The largest average length ratio recorded in this range was ~2.8, which occurred on the 2 μm comb structure. In comparison, the length ratio of cell nuclei on the blanket tantalum surface was 1.5 ± 0.4. Nuclei on the 50 and 100 μm comb structures did not exhibit significant elongation with length ratios of ~1.5. The relationship between the axis length ratio and the percentage distribution of cells aligned within 10° of the line axes is shown in Figure 5d. Results showed that as more cell nuclei aligned to the line axes, the average elongation of cell nuclei also increased.

### 3.3. Nanometer Scale Morphology Analyses

#### 3.3.1. Cells on Individual Comb Structures

**Figure 6:**
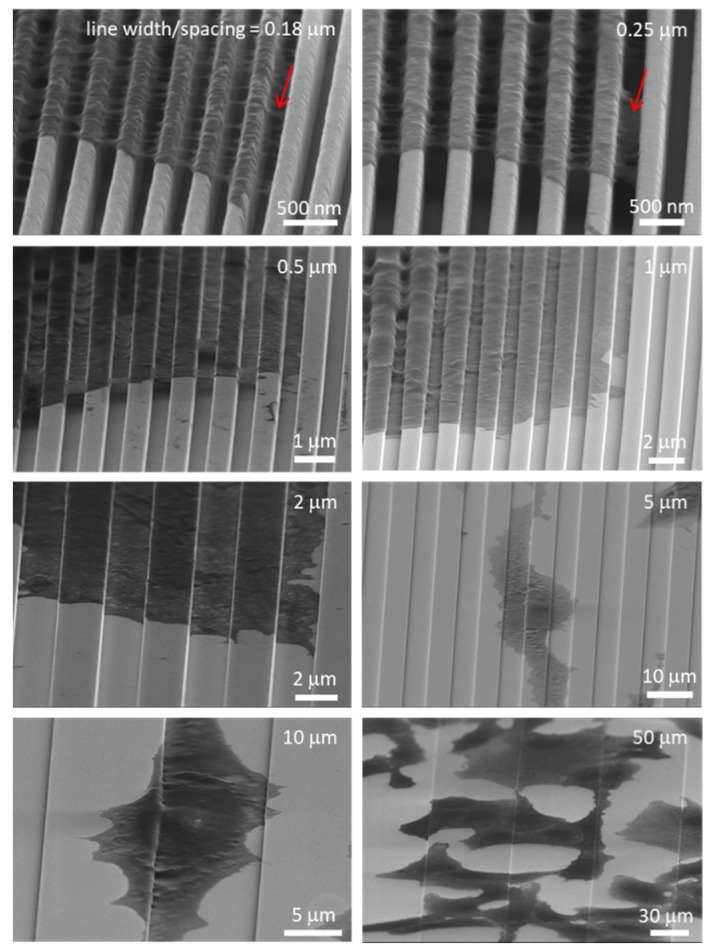
SEM micrographs 70° tilted of cells on comb structures with line widths of 0.18–50 μm. Two distinct types of cell adhesion morphologies are observed: Type 1—Adherent cells on 0.18 μm and 0.25 μm structures only contacted the top portion of lines but did not fill the trench gaps, and Type 2—Cells on comb structures with line widths larger than 1 μm exhibit conformal surface coverages. Both morphology types were observed for adherent cells on the 0.5 μm comb structures. All cells were incubated on the structures for 24 h.

**Figure 7:**
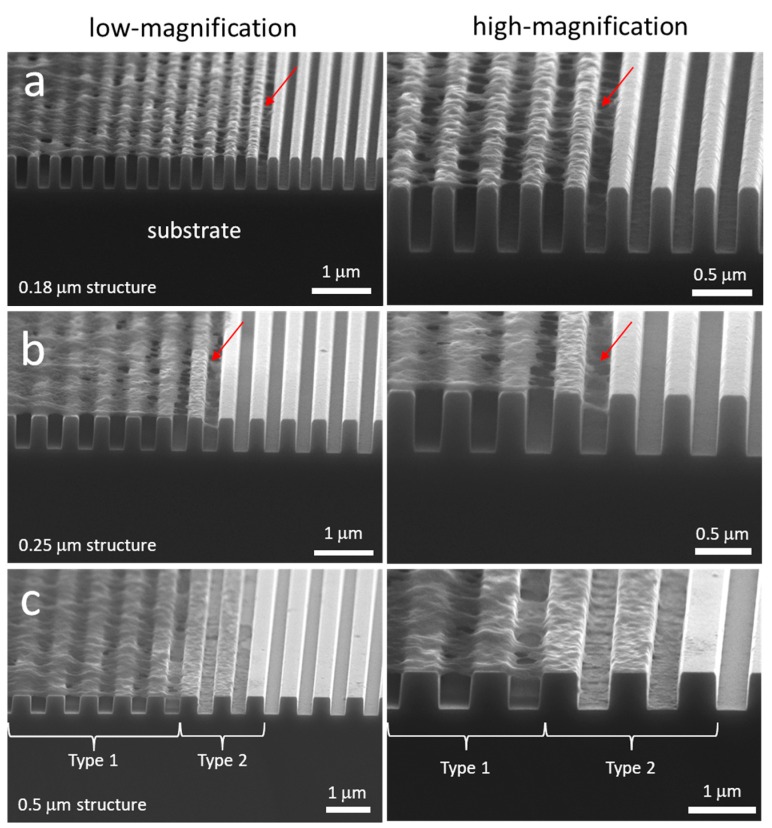
SEM micrographs 70° tilted of cross-sectioned cells on comb structures with line widths of (**a**) 0.18 μm, (**b**) 0.25 μm, and (**c**) 0.5 μm. Two distinct types of cell adhesion morphologies are observed: Type 1—Adherent cells on 0.18 μm and 0.25 μm structures only contacted the top portion of lines but did not fill the trench gaps; and Type 2—Cells exhibit conformal surface coverages. Both morphology types were observed for adherent cells on the 0.5 μm comb structures. All cells were incubated on the structures for 24 h. Cell concentration was ~5 × 10^5^ cells/mL.

Nanometer-scale cell morphology in the three cell alignment regions (i)–(iii) were characterized using high-resolution field-emission SEM techniques. Field-emission SEM was chosen because of the resolution that could be achieved, i.e., smaller than 2 nm. Typical 70° tilted SEM micrographs of cells on 0.18, 0.25, 0.5, 1.0, 2.0, 5.0, 10, and 50 μm-wide line comb structures are shown in Figure 6. These high-angle tilted micrographs captured the three-dimensional morphology of pseudopodia at the periphery of the cells. Micrographs show nanometer-scale pseudopodia spreading in directions parallel and perpendicular to the line axes on the 0.18 and 0.25 μm comb structures. Short pseudopodia filament-like structures that were oriented perpendicular to the line axes are highlighted with red arrows. Some have diameters in the order of ~50 nm and appear to be floating on the patterned structures. To verify this morphology, cells were cross-sectioned by micro-cleaving and inspected using SEM. Typical 70° tilted micrographs of cross-sectioned cells on 0.18 and 0.25 μm comb structures are shown in Figure 7a,b, respectively. Peripheral pseudopodia, highlighted with red arrows, projected across the trench and adhered to adjacent sidewalls. These structures adhered to locations ~80 nm below the top surface and did not contact the trench bottoms—they formed bridges across the trenches. This type of morphology (denoted as Type 1) was also observed away from the periphery of the cell (~2.5 μm) on the 0.5 μm line comb structure (Figure 7c). On the 0.5 μm line comb structure of the periphery, the cells adhered to both sidewalls and trench bottom. This type of cell behavior, denoted as Type 2, was the only morphology observed in comb structures with line widths larger than 1 μm (Figure 6).

**Figure 8:**
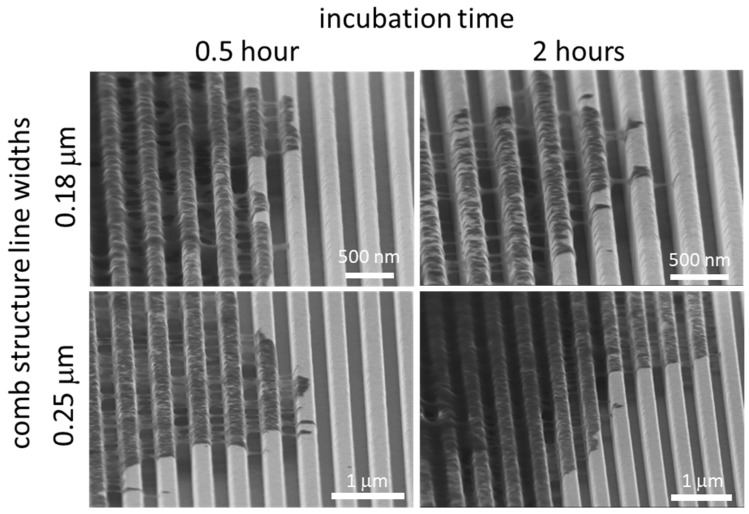
Typical SEM micrographs show Type 1 cell morphology was observed on 0.18 and 0.25 μm comb structures after 0.5 and 2 h of incubation.

To determine whether these phenomena occurred during the initial spreading process or only after 24 h of incubation, high-resolution SEM was used to probe the cells on 0.18 and 0.25 μm comb structures after 0.5 and 2 h of incubation. Micrographs in Figure 8 clearly show that the Type 1 pseudopodia morphology occurred as early as 0.5 h post-deposition. The majority of the filament-like structures that were observed bridged across the trenches and adhered to the adjacent sidewalls. Some filament-like structures migrated up the trench sidewalls and covered the line’s top surfaces. No cellular material was observed contacting the bottom of the trenches. Additional micrographs of adherent cells on 0.18 and 0.25 μm comb structures after 0.5 h of incubation are shown in Figures S3a and S3b, respectively. The low magnification images of adherent cells reveal that they were not fully spread and had a thick interior region, though the morphology of the peripheral part of the cell was consistent with the images from longer term incubation (Figure 7 and Figure S3c). This morphology does not appear to be the result of the cell contraction process during reshaping as described by others [18].

The influence of surface topographic parameters, such as trench depth (D), trench width (T), and line width (L), on cell behavior and morphology was also investigated by other researchers [16,37,64,65,66,67,68]. Loseberg et al. [66], Lamers et al. [65], and Ventre et al. [64] showed that fibroblast and osteoblast cell alignment is generally induced by line structures separated by trenches at least 35 nm deep. Lamers and colleagues [65,68], and Toworfe et al. [67] theorized that trenches less than 35 nm deep fill with serum proteins and “smooth” the patterned structure. Line width has also been shown to control cell morphology [16,64,65,66,67,68]. Loseberg et al. [66] and Lamers et al. [65] further reported that line and trench widths larger than 80–100 nm are required to successfully align cells. Our work is consistent with these findings, with cell alignment occurring on patterned surfaces having line widths greater than 180 nm and trench depth of ~700 nm. Trench spacing is also important. Depending on the spacing between lines, cells may conformally coat the trench or bridge across the trench [64]. Epithelial cells deposited on patterns consisting of 330 nm-wide and 150 nm-deep trenches separated by 70 nm lines anchored on the lines and were not able to adhere to the bottom of the trench [16]. In a recent review by Ventre et al. [64], cells are said to “float” on dense patterned structures without contacting the bottom of trenches when line and trench widths are smaller than 100 nm and trench depths are larger than 40 nm. These conditions are also consistent with the observations shown in Figure 6 where cells on comb structures with trench and line widths smaller than 250 nm did not contact the trench bottom.

As trench width increases, cells begin to descend into the trenches [16,45,64] leading to a conformal coating of the surface by the cell. Ventre et al. [64] suggested that a general topographic structural requirement for cells to descend into a trench includes trench widths larger than 1 μm and line widths larger than 100 nm. Zahor et al. [45] showed elongated cells prefer occupying 5 μm-wide trenches rather than the top of lines. Such preferential adhesion is consistent with the results observed in this work (Figure 4 and Figure 6), where the majority of cells deposited on the 10 μm comb structures elongated and descended into the trenches.

#### 3.3.2. A Single Cell Adhered on Multiply Structures Simultaneously

**Figure 9:**
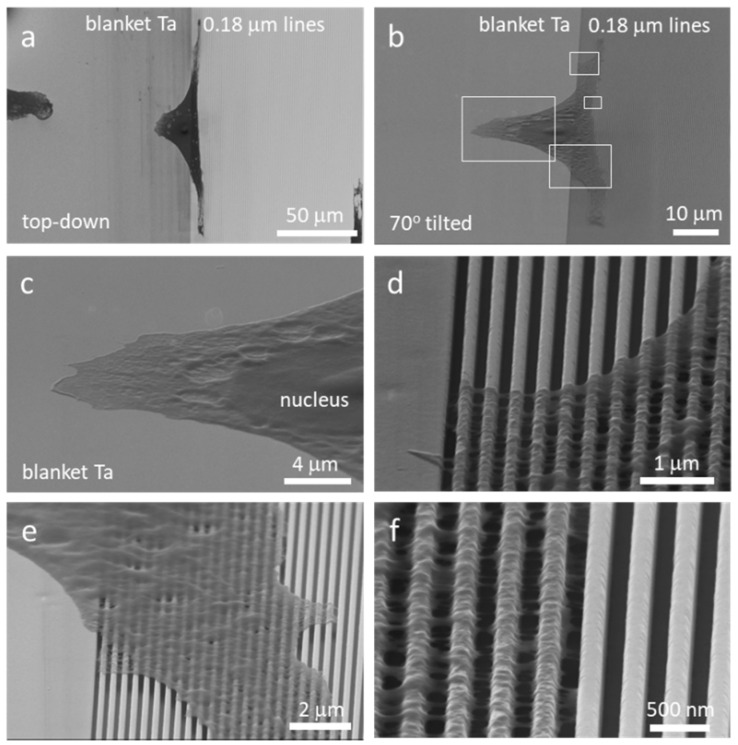
(**a**) Top-down and (**b**) 70° tilted SEM micrographs of an adherent cell partially on the blanket Ta region and on 0.18 μm structures. Portion of cells rested on the blanket tantalum (Ta) region (**c**–**e**) showing regions where cell adhered on blanket Ta and comb structure. (**f**) A high magnification image of cell pattern structure.

Cell morphology can be influenced by the surface topography of the substrata on which cells can adhere [54,64,69,70,71]. However, previous observations were based on cells adhered to a uniformly textured surface. It remained unclear how a cell would behave when exposed to a non-uniform textured surface having multiple structures with different geometries. Low magnification top-down and 70° tilted SEM micrographs of cells incubated for 24 h on a smooth blanket tantalum surface and a 0.18 μm comb structure are displayed in Figure 9a,b, respectively. The micrographs indicate that approximately half of the cell and its nucleus adhered to the smooth surface, while the rest of the cell adhered to the comb structure. This is thought to be the first study revealing how mammalian cells can have different morphologies within the same cell when they simultaneously adhere to two different engineered structures. Images showed that cellular materials on the flat surface were spread without preferential orientation. In contrast, the portion of the cell that rested on the patterned line structures elongated and aligned parallel to the line axes. Furthermore, some of the cellular material on the flat surface, adjacent to the comb pattern boundary, appeared to have stretched along the line axes. This may indicate that mechanical stresses were transmitted across the cell; however, the elongation of the cell may have been hindered by the portion of the cell that was anchored on the flat surface. Selected high magnification 70° tilted SEM micrographs of this cell are shown in Figure 9c–f. These micrographs show that the portion of the cell on the comb structure exhibited a Type 1 cell morphology with most cellular materials adhering to the top surfaces of the lines. Nanometer-scale pseudopodia were observed to bridge across the trench gaps as illustrated in Figure 7. These results demonstrate that cells regulated their morphology at a localized level. Additional micrographs of a cell that was incubated on the 0.25 μm comb structures for 0.5 h is shown in Figure S4. The majority of this cell and its nucleus adhered to the flat tantalum surface, while the rest adhered to the comb structures. Type 1 spreading is clearly visible in the comb structure area. However, this cell did not elongate due to the short incubation time.

#### 3.3.3. Long-Stranded Pseudopodia Structures

**Figure 10:**
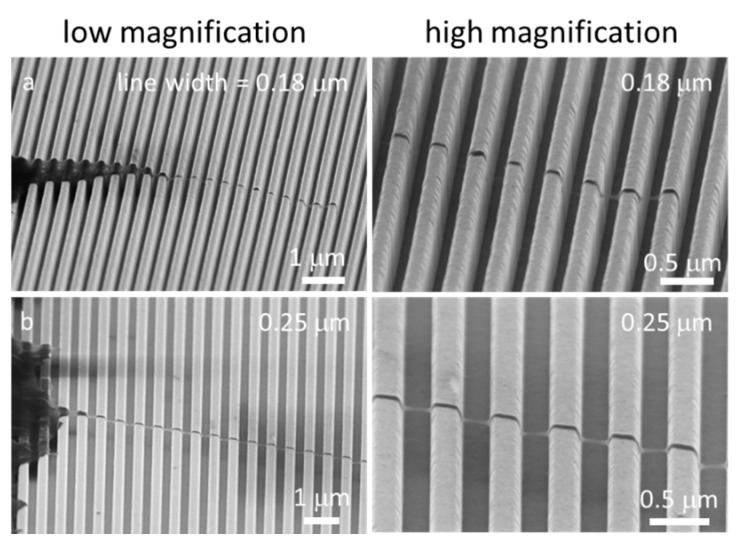
Typical SEM micrographs of adherent cell pseudopodia on (**a**) 0.18 μm and (**b**) 0.25 μm comb structures under low and high magnifications. Type 1 characteristics were observed in all filaments where they wrapped around the top portions of the lines.

In addition to the short pseudopodia filament-like structures shown in Figure 7, micrometer-long single-stranded pseudopodia were also observed. The diameter of these cellular structures was in the order of 50 nm. Representative low- and high-magnification micrographs of these cytoplasmic projections on 0.18 and 0.25 μm comb structures are displayed in Figure 10a,b, respectively. Results showed that these long-stranded pseudopodia bridged across trench gaps on the 0.18 and 0.25 μm comb structures without contacting the trench bottom. This demonstrated that the morphology of long cytoplasmic projections was similar to those short pseudopodia filament-like structures observed at the cell periphery, as shown in Figure 8. Both exhibited a Type 1 structure when cells adhered to comb structures with line widths of 0.18 to 0.25 μm. In addition, Figure 10 shows that the long single-stranded pseudopodia morphology did not change with the distance from the cell periphery.

### 3.4. Possible Cell Alignment Mechanisms

One potential contributing factor to the elongated cell morphology shown in Figure 4, Figure 5, Figure 6 and Figure 7 could have been due to larger mechanical constraints on cell spreading in the direction perpendicular to the lines. When cells spread perpendicular to the line axes, cellular components must overcome the physical constraints by crawling up and down trench sidewalls. These surface topographic features reduce the cell spreading velocity and motility in the perpendicular direction. In contrast, there was no topographic-induced constraint in the direction along the line axes where cells could spread readily. Notably, the amount of mechanical constraint perpendicular to the line axis was pattern-dependent. The number of sidewalls per unit length in the direction perpendicular to the line axes increased with smaller comb structure line widths. Hence, the total distance that cells traveled on dense line comb structure was larger than distances travelled on flat surfaces without any sidewalls, or on comb structures with few sidewalls. Therefore, the amount of cell elongation and alignment was larger on dense line patterns than on flat surfaces. For example, a simple mathematic calculation can show that the total distance a cell spreads perpendicularly across the 1 μm comb structures is ~70% larger than the total distance a cell spreads on the flat surfaces. The slight reduction in cell nuclei alignment performance on 0.18 to 0.5 μm comb structures may seem contradictory to the aforementioned hypothesis; however, this discrepancy may be explained by the different cell-spreading mechanisms (Type 1 vs. Type 2). Figure 7 and Figure 8 show that during the Type 1 spreading process, pseudopodia structures only contacted the top ~80 nm of the trench walls and then bridged across the gaps between adjacent lines. The pseudopodia structures did not cover the entire trench sidewalls and did not contact the trench bottom surfaces. Furthermore, the total distance pseudopodia travelled on the 0.18 μm comb structures was actually ~18% smaller than that travelled on the 1 μm comb structure, but still ~44% greater than that travelled on flat surfaces. Hence, although the cell nuclei on 0.18 μm structures were not as well-aligned as those on the 1 μm comb structure, they performed better than cells on blanket flat surfaces. The cell alignment mechanism proposed here is also consistent with observations by Zhou et al. [41], who reported increases in cell alignment toward the line axes when the cell membrane penetration depths into grooves was larger.

One interesting aspect of the Type 1 cell spreading mechanism is the empty trench space near the cell periphery, which is in the order of hundreds of nanometers wide. These openings create free-standing cell structures that allow a greater surface area to make contact with the surrounding environment, in contrast to cells with the Type 2 spreading mechanism in full contact with the substrata. It is unclear whether these openings produced by the Type 1 morphology could give rise to greater access for contact with nano-particles, or in the context of surgical implants, whether Type I spreading could increase the susceptibility to infection.

In summary, our results demonstrated that patterned tantalum coatings can be used to manipulate cell alignment and morphology. These coatings can potentially be applied to porous scaffold structures as a method to improve matrix material bioactivity or enhance bone regeneration in surgical implants.

## 4. Conclusions

Adherent mammalian cells (Vero) were elongated on tantalum-coated comb structures with line/trench widths in the range of 0.18 to 10 μm. As much as 77% of the cell nuclei aligned with the line axes. Cell pseudopodia exhibited two types of morphologies that depended on the line and trench widths. First, when widths were smaller than 0.5 μm, nanometer-scale pseudopodia structures bridged across the trenches without contacting the bottom surfaces. Second, cells conformed completely with the surface topology on comb structures having wider line spacing. Results also revealed that individual cells can exhibit multiple morphologies when simultaneously exposed to varying engineered features.

## Figures and Tables

**Table 1:** Results of cell alignment performance on various substrate materials and surface pattern designs.

Reference	Cell Type	Substrate	Line Width Range (μm)	Trench Width Range (μm)	Maximum Alignment Line/Trench Width (μm)
[44]	Human corneal epithelial cells	Silicon oxide	0.07–1.9	0.3–2.1	0.85/1.15
[54]	Osteoblast-like cells (MG63)	Silicon	0.09–0.5	0.09–0.5	0.15/0.15
[48]	HeLa cells	Polydimethylsiloxane	2–30	1.5–3.0	2/2
[38]	Human neural stem cells	Polydimethylsiloxane	5–20	5–60	5/5
[37]	Human mesenchymal stem cells	Polystyrene stripes	5–1000	5–1000	20/20
[40]	Adult neural stem cells	Poly-d-lysine with Printed laminin strips	30	30–170	30/30

**Table 2:** Data summary of number of cells inspected (n), percent population of cells with 10° > ϕ > −10° of the line axis, and axis length ratio (L/S). The culture media initial cell concentration used was ~0.5 × 10^5^ cells/mL. Data spreads correspond to one standard deviation.

Structure	Line (L)/Trench (T) Width (μm)	Inspected Comb Structure Area (mm^2^)	Number of Cells Sampled (n)	Coverage (cell/mm^2^)	L/S	% of Population Aligned ±10^°^ from Lines
1	0.18	1.8	281	156	2.2 + 0.7	63.0 + 1.4
2	0.25	1.8	171	95	2.3 + 0.8	55.6 + 4.1
3	0.5	1.8	235	131	2.1 + 0.6	53.2 + 7.4
4	1	1.8	197	109	2.7 + 0.9	77.7 + 2.0
5	2	1.8	179	99	2.8 + 1.4	68.7 + 4.9
6	5	1.8	238	132	2.4 + 1.2	68.0 + 6.2
7	10	1.8	159	88	2.3 + 0.8	71.7 + 8.6
8	50	1.8	337	187	1.6 + 0.3	18.7 + 7.7
9	100	6.6	947	143	1.5 + 0.4	17.4 + 0.3
10	blanket Ta	1.8	303	168	1.5 + 0.4	8.6 + 5.5

## Supplementary Materials

The following are available online at http://www.mdpi.com/2072-666X/9/9/464/s1, Figure S1: Copper stripped comb pattern structures, Figure S2: Fluorescence confocal micrographs of adherent cells on 0.25, 0.5, 1.0, and 10 μm comb structure after 24 hours of incubation, Figure S3: (**a**) 70° tilted SEM micrographs of an adherent cell on 0.18 μm comb structure after 0.5 hour of incubation; (**b**) 70° tilted SEM micrographs of an adherent cell on 0.25 μm comb structure after 0.5 hour of incubation; (**c**) 70° tilted SEM micrographs of an adherent cell on 0.18 μm comb structure after 9 hours of incubation, Figure S4: 70° tilted SEM micrographs of a cell simultaneously adhered on flat surfaces and 0.25 μm comb structure after 0.5 hour of incubation.
