# Supplementary material for: Nanoscale-Textured Tantalum Surfaces for Mammalian Cell Alignment
Source: Micromachines (Basel). 2018 Sep 13;9(9):464. doi: 10.3390/mi9090464 (PMC6187670; doi:10.3390/mi9090464)
Supplement: Supplementary file 1 [file micromachines-09-00464-s001.pdf]

Supplementary Materials: Nanoscale-Textured Tantalum Surfaces for Mammalian Cell Alignment

Hassan I. Moussa, Megan Logan, Kingsley Wong, Zheng Rao, Marc G. Aucoin and Ting Y. Tsui

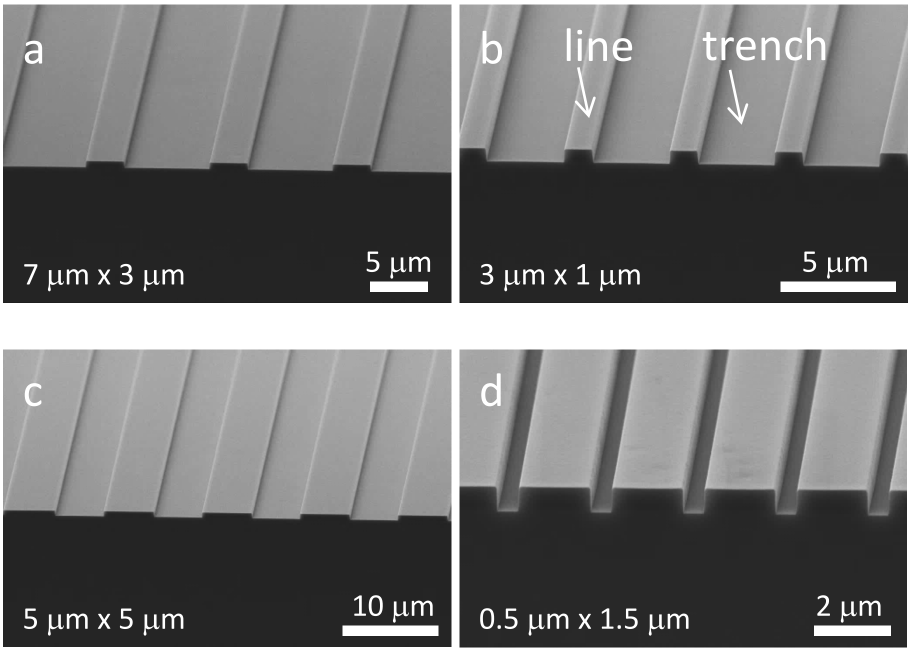

Figure S1. Copper stripped comb pattern structures.

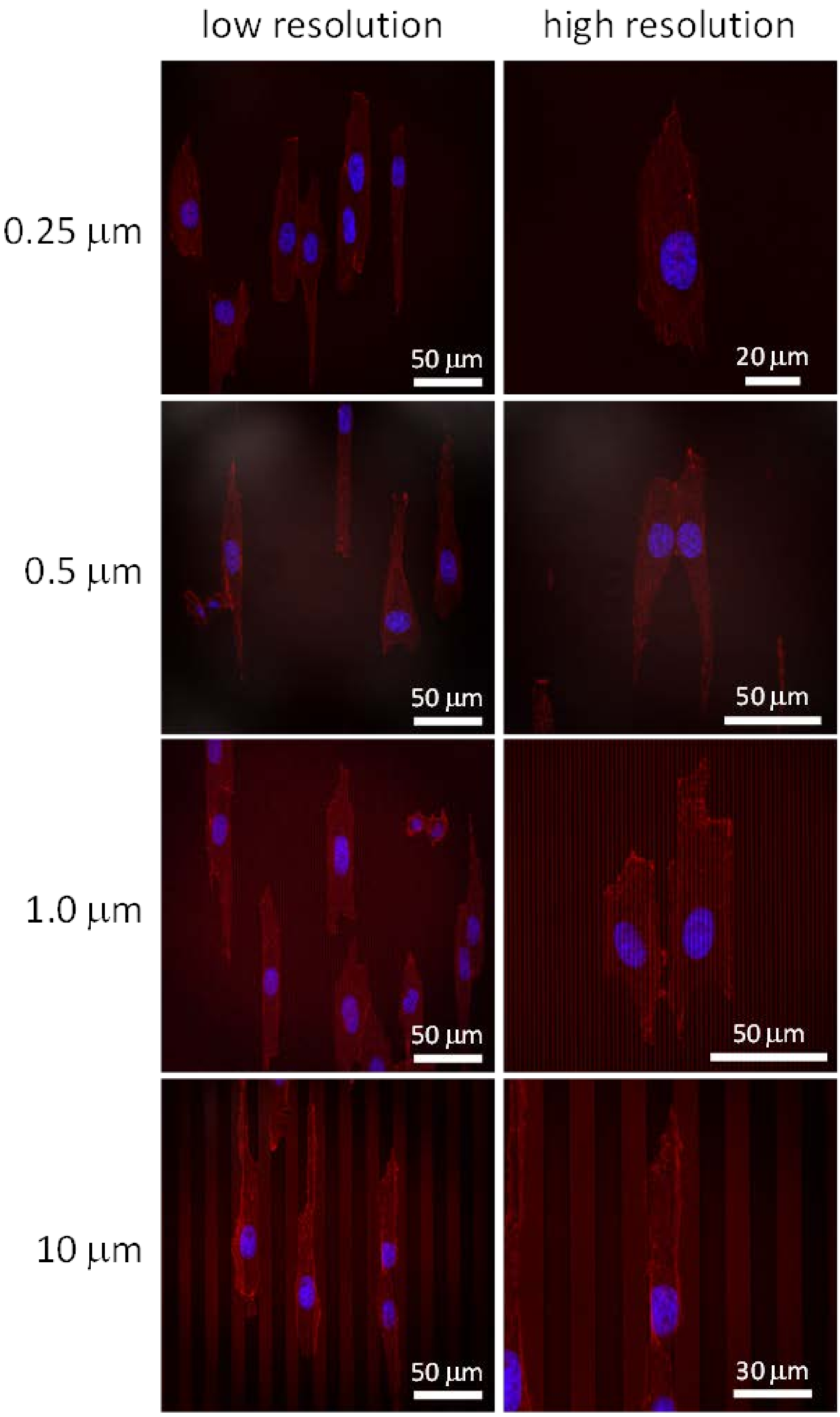

Figure S2. Fluorescence confocal micrographs of adherent cells on 0.25, 0.5, 1.0, and 10 μm comb structure after 24 hours of incubation.

0.5 hour incubation on 0.18 µm comb structure

(**a**)

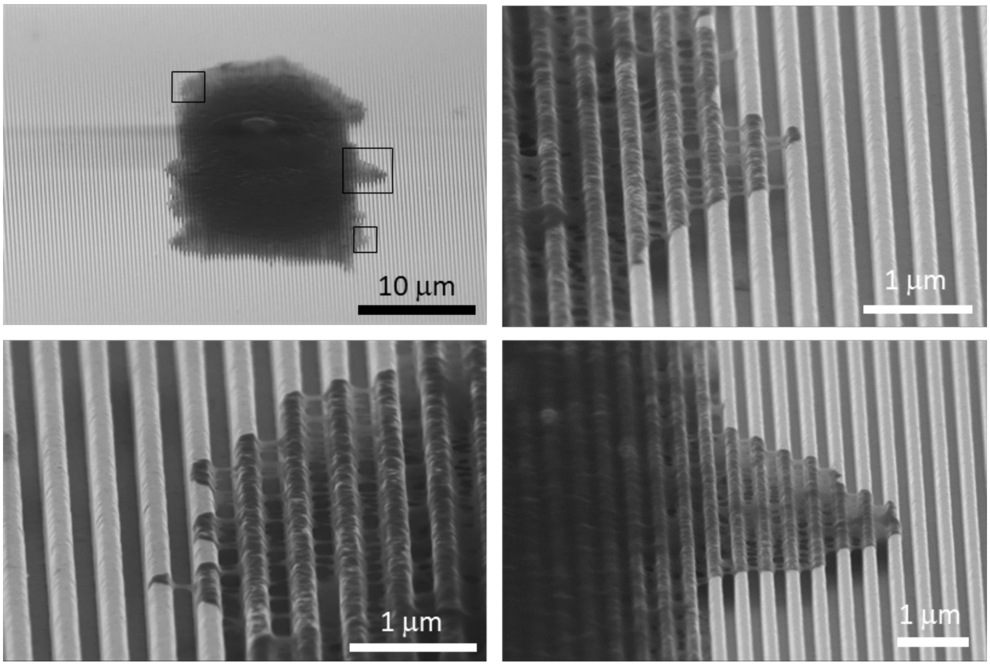

0.5 hour incubation on 0.25 µm comb structures

(**b**)

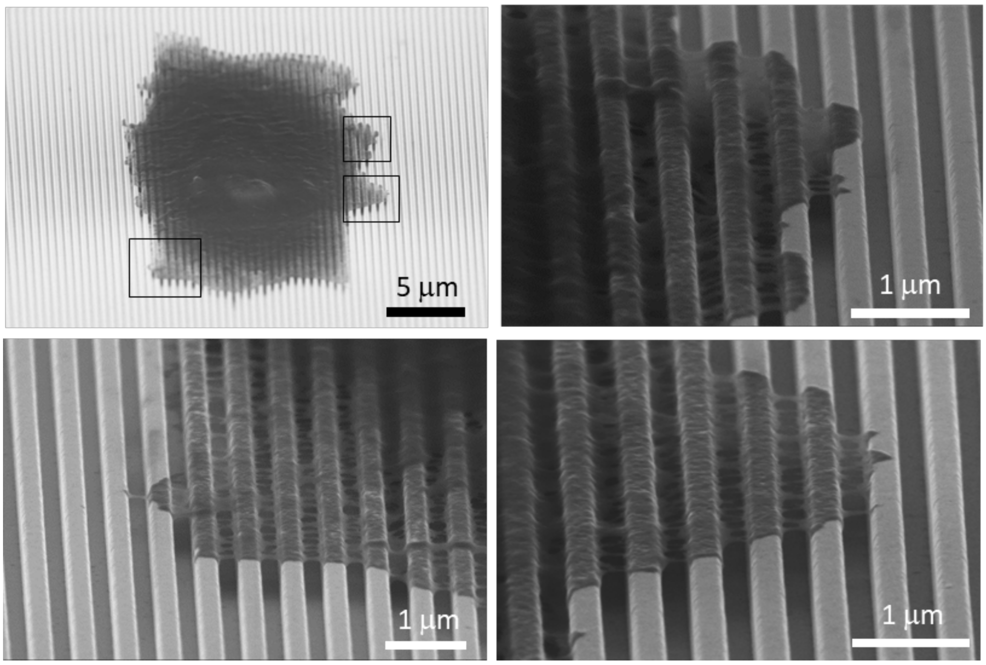

(c)

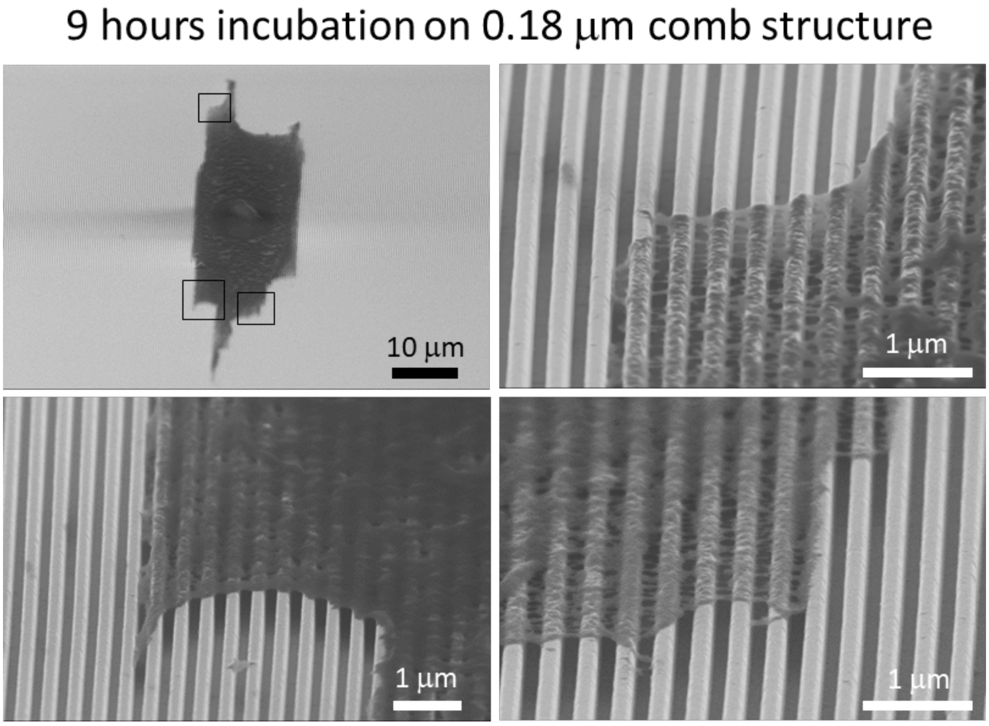

Figure S3. (**a**) 70° tilted SEM micrographs of an adherent cell on 0.18 μm comb structure after 0.5 hour of incubation. (**b**) 70° tilted SEM micrographs of an adherent cell on 0.25 μm comb structure after 0.5 hour of incubation. (**c**) 70° tilted SEM micrographs of an adherent cell on 0.18 μm comb structure after 9 hours of incubation.

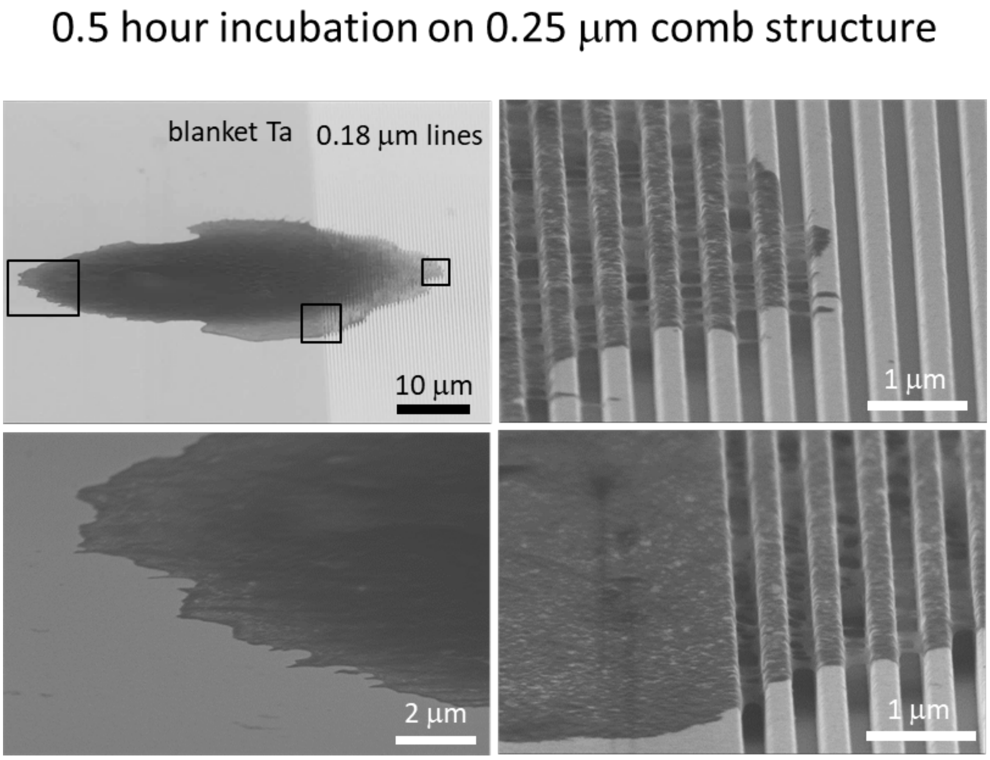

Figure S4. 70° tilted SEM micrographs of a cell simultaneously adhered on flat surfaces and 0.25 μm comb structure after 0.5 hour of incubation.
